# Neighborhood Perceptions Among Pregnant African American Women in St. Louis, Missouri, Before and After the Shooting of Michael Brown

**DOI:** 10.1089/heq.2019.0125

**Published:** 2020-08-19

**Authors:** Rachel G. Tabak, Karishma Furtado, Cynthia D. Schwarz, Debra Haire-Joshu

**Affiliations:** ^1^ Prevention Research Center in St. Louis, Brown School at Washington University in St. Louis, St. Louis, Missouri, USA.; ^2^ Center for Diabetes Translation Research, Brown School, Washington University in St. Louis, St. Louis, Missouri, USA.; ^3^ Center for Obesity Prevention and Policy Research, Brown School, Washington University in St. Louis, St. Louis, Missouri, USA.; ^4^ Washington University School of Medicine, Washington University in St. Louis, St. Louis, Missouri, USA.

**Keywords:** environment, pregnancy, socioeconomic factors

## Abstract

**Purpose:** This study aims to examine perceptions of neighborhood quality and safety before and after the death of Michael Brown and the unrest that followed.

**Methods:** In this secondary analysis of baseline data from one site in The Lifestyle Interventions for Expectant Moms (LIFE-Moms) Consortium, pregnant African American women in the St. Louis region completed a survey of neighborhood perceptions. Logistic regression was used to explore associations between perceptions among those completing baseline surveys and entering the study before and after August 9, 2014 (range: 2012–2015), adjusted for demographic characteristics.

**Results:** Of 267 participants, half (*n*=134) completed the survey after August 9, 2014. Thirty-four percent of participants completing the survey after this date felt “The crime rate in my neighborhood makes it unsafe to go on walks during the day” compared with 21% of those completing the survey before (adjusted odds ratio=2.0, 95% confidence interval: 1.1–3.7). There were no consistently significant differences in demographic characteristics or in the remaining 16 neighborhood items.

**Conclusions:** This study is an example of how an unexpected shift in the community context in the wake of a profound event may impact health behaviors and outcomes in a measurable way.

**Clinical Trials Registration:** NCT01768793.

## Introduction

The discussions of health equity and social determinants of health in the public health literature are more prominent and articulate a critical discourse around the many influences on health and health outcomes.^1–4^ Inequalities in neighborhood environments are hypothesized to drive these health disparities, and direcly impair public health efforts to achieve health equity.^5^ To “optimize the conditions in which people are born, grow, live, work, learn and age,”^1^ public health research must build an empirical understanding of how environments and environmental social contexts impact individuals and their communities and lead to inequities and disparities.

A large body of research reminds us that the everyday manifestation of racism in the environments that individuals traverse takes a toll on the body.^6,7^ The relationships between local and neighborhood environments and health outcomes are important components of these determinants. For example, the For The Sake of All report published in the summer of 2014 found that residents in the St. Louis, Missouri area “separated by only few miles have up to an 18-year difference in life expectancy.” The report also highlighted disparities in access to resources such as financial services and healthy food and stated: “Because of considerable residential segregation in St. Louis, many areas with high African American populations are also areas with concentrated poverty and poor health.”^8^ While these findings are unique to St. Louis, health disparities influenced by segregation are widespread in the United States.^9^

Around the time of the publication of the For The Sake of All report, other events in St. Louis highlighted profound inequities in the community.^10^ On August 9, 2014, Michael Brown was shot and killed by police officer Darren Wilson in the St. Louis suburb of Ferguson, Missouri. The death of Michael Brown and the civil unrest that followed have come to symbolize a deeply fractured relationship between African Americans and law enforcement and the racial lines dividing access to opportunity. The peaceful memorials that began on August 9th became more agitated by the evening of the 10th. Looting and vandalism began that night with multiple buildings set on fire. Approximately 150 police officers in riot gear were deployed in response. That night and over the following days and weeks, the unrest continued, with police response often escalating to the use of chemical weapons, smoke bombs, flash grenades, and rubber bullets. The city, statewide, and national response to the unrest received widespread, national media attention.^11,12^

Ferguson quickly became international shorthand for the militarization of police response to mass demonstration; the long history of systematic racism embedded within our institutions, especially the justice system; the deep racial divides between community and law enforcement; and profound civil unrest at all of the above. Survey research in the St. Louis County demonstrated negative impacts of these events on perceptions of the police and mental health among African American residents^13,14^; similar results have been seen at a national level.^15^ In light of these findings, this study seeks to examine how perceptions of neighborhood quality and safety differed before and after the death of Michael Brown and the unrest that followed.

When explored at the individual level, understanding perceptions of the neighborhood environment and how local events might shape them is critical, as these perceptions are hypothesized to influence health and a number of health behaviors, including physical activity.^16–18^ Understanding an individual's environmental and social context is particularly important when designing, testing, and implementing interventions to promote health behaviors. For example, Alang et al. proposed five intersecting pathways by which police brutality can influence health disparities (e.g., adverse physiological responses that increase morbidity and racist public reactions that cause stress), although they identify a lack of empirical literature.^10^

To contribute to the picture of how officer-involved shootings and the resulting unrest may contribute to health, the current study explores baseline data from a large trial investigating an intervention to promote healthy gestational weight gain among African American women with low socioeconomic status in the St. Louis area. Of relevance to the above discussion, the time frame for recruitment and baseline assessment spanned the period before and after the death of Michael Brown on August 9, 2014, and the baseline survey included a measure of neighborhood perceptions related to health that has been used in numerous other settings.^19–22^ The objectives therefore are to explore neighborhood perceptions among the entire study population and then to contrast perceptions among those completing the survey before with those completing the survey after August 9, 2014. A metaobjective is to examine one way (i.e., analytically) a study can remain responsive to the dynamic social context in which it is set.

## Methods

### Participants and procedures

This is a secondary analysis of baseline data from one site in The Lifestyle Interventions for Expectant Moms (LIFE-Moms) Consortium.^23^ This consortium was designed to determine whether various behavioral and lifestyle interventions reduce excessive gestational weight gain among pregnant women with overweight or obesity as well as adverse outcomes among mothers and their babies; details of the consortium are published elsewhere.^24,25^

The current study includes the 267 participants from the Washington University in the St. Louis site. All women were recruited through obstetrics clinics at a single medical center (Barnes-Jewish Hospital and Washington University School of Medicine in St. Louis, Missouri), whose catchment area includes most of the St. Louis area. To be included in the study, women met the following eligibility criteria: (1) age 18–45 years, (2) identified as African American, (3) body mass index 25.0–45.0 kg/m^2^ measured at the initial visit during the first trimester, (4) singleton viable gestation at or before 15 0/7 weeks (established by date of last menstrual period if it was within 5 days of first trimester ultrasound dating or by ultrasound itself), and (5) socioeconomically disadvantaged determined by Medicaid status or home zip code associated with a median household income below the poverty level. Recruitment occurred from October 2012 to March 2016. All women provided written informed consent before their participation in this study, which was approved by the Institutional Review Board of Washington University in St. Louis.

While the current study reports only on baseline measures, the full study included a randomized trial to evaluate an intervention designed to extend from pregnancy through 18 months postpartum. The results of the trial on gestational weight gain and postpartum weight retention, as well as descriptions of the intervention, measures, and procedures are available elsewhere.^23–25^ The data for the current study come from those collected at 15 weeks of gestation (baseline).

### Measures

Participants completed surveys and had their height and weight measured at the Washington University School of Medicine in St. Louis' clinical research unit. Survey measures included demographic characteristics as well as the Physical Activity Neighborhood Environment Survey (PANES) questionnaire (dependent variable in the current study). PANES is a 17-item survey designed to assess perceptions of the neighborhood environment, particularly as they relate to attributes hypothesized to influence physical activity.^19^

This measure includes participant report of the main type of housing in her neighborhood (e.g., apartment, town house, single family home), which is a proxy assessment of residential density. Housing type was recoded as detached single-family homes and multifamily homes (e.g., apartment, town house, single family home), as has been done in previous studies.^19–21^ Participants reported on access to destinations such as shops and transit stops; infrastructure such as the presence and quality of sidewalks, bicycle facilities, and free or low-cost recreation facilities (e.g., parks, public swimming pools); the aesthetic qualities and social environment of the neighborhood; street connectivity; and neighborhood safety (i.e., traffic and daytime and nighttime crime).

Response options were based on a Likert-type scale: 1, strongly disagree; 2, somewhat disagree; 3, somewhat agree; 4, strongly agree; don't know/not sure. Consistent with previous literature, responses were dichotomized as “strongly agree/agree” versus “disagree/strongly disagree.” Participants responding “don't know/not sure” were coded as missing. The independent variable for this study was determined by when the baseline survey was completed (i.e., before or after August 9, 2014; no baseline assessments occurred on this date).

### Statistical analysis

This study is nonexperimental. Perceptions of the neighborhood environment were explored, looking at the percent of participants in the whole sample agreeing/disagreeing with each statement. We then compared aggregated data across two groups of participants: (1) those completing the baseline survey before August 9, 2014, and (2) those completing it after August 9, 2014. Independent samples *T*-tests were utilized for continuous variables (e.g., age) and chi-square (*χ*^2^) or Fisher exact test for categorical variables (e.g., binary perceived neighborhood attributes) to compare participants completing the baseline survey before versus after August 9, 2014.

Logistic regression models were used to determine the crude odds ratio for the association between survey group (baseline survey before/after August 9, 2014—independent variable) and binary neighborhood attribute (dependent variables). These models were then adjusted for demographic factors: maternal age, income, level of education, and marital status.

## Results

Of the 267 participants, half (*n*=133) completed the survey before and half (*n*=134) completed the survey after August 9, 2014. Among participants completing the survey before August 9, 2014, the average length of time before the date was 267 days (standard deviation [SD]: 162 days; range 2–627 days); for those completing the survey after, the average length of time after the date was 211 days (SD: 108 days range 3–391 days).

Demographic characteristics for the whole sample and separated based on when the survey was completed are shown in Table 1; no differences were observed between those taking the survey before and those taking the survey after August 9, 2014. Mean age was 25.8 (SD=5.0) years and mean body mass index was 32.3 (SD=5.0). Consistent with inclusion criteria, all mothers identified as African American. Ninety-two percent (*n*=236) of the mothers were insured through Medicaid. Nearly 40% (*n*=103) of the sample reported an income of less than $5000 per year and 73% (*n*=193) reported an income of less than $15,000 per year. Just under half (*n*=114, 43%) were married or living with a significant other. Only 4.5% of mothers (*n*=12) reported owning a single-family house or town house or condo; 62% (*n*=164) reported renting, and 34% (*n*=90) reported living in the home of their parents or other adults.

**Table 1.** Baseline Characteristics of the St. Louis, Missouri, Mothers Participating in the LIFE-Moms Trial for the Total Sample and for Those Who Completed Their Baseline Survey Before and Those Who Completed Their Baseline Survey After August 9, 2014, the Date Michael Brown Was Shot

	Total	Before	After	p
Maternal age (years)^a^	25.84 (5.00)	25.85 (5.36)	25.84 (4.64)	0.98
Baseline body mass index (kg/m^2^)^a^	32.30 (5.03)	32.22 (4.91)	32.38 (5.16)	0.80
Body mass index category^b^				
Healthy/overweight	95 (35.6)	42 (31.6)	53 (39.6)	
Obese	172 (64.4)	91 (68.4)	81 (60.5)	0.17
Receive Medicaid^b^	236 (92.2)	114 (90.5)	122 (93.9)	0.32
Maternal education^b^				
Less than high school	54 (20.2)	28 (21.1)	26 (19.4)	
High school graduate	110 (41.2)	53 (39.9)	57 (42.5)	
Some college	83 (31.1)	45 (33.8)	38 (28.4)	
College graduate	20 (7.5)	7 (5.3)	13 (9.7)	0.46
Income level^b^				
<$5000	103 (39.0)	56 (43.1)	47 (35.1)	
$5000–$9999	48 (18.2)	22 (16.9)	26 (19.4)	
$10,000–$14,999	42 (15.9)	14 (10.8)	28 (20.9)	
≥$15,000	71 (26.9)	38 (29.2)	33 (24.6)	0.11
Marital status^b^				
Not married	144 (53.9)	65 (48.9)	79 (59.0)	
Separated/widowed/divorced	9 (3.4)	6 (4.5)	3 (2.2)	
Not married and living with significant other	85 (31.8)	43 (32.3)	42 (31.3)	
Married	29 (10.9)	19 (14.3)	10 (7.5)	0.16
Current living situation^b^				
Own single-family house/town house/condo	12 (4.5)	8 (6.1)	4 (3.0)	
Rent	164 (61.7)	75 (56.8)	89 (66.4)	
Live in the home of your parents or other adults	90 (33.8)	49 (37.1)	41 (30.6)	0.20

^a^ Data are mean±SD; *p*-value based on *t* test.

^b^ Data are *n* (%); *p*-value based on *χ*^2^ or Fisher exact test.

SD, standard deviation.

**FIG. 1.**
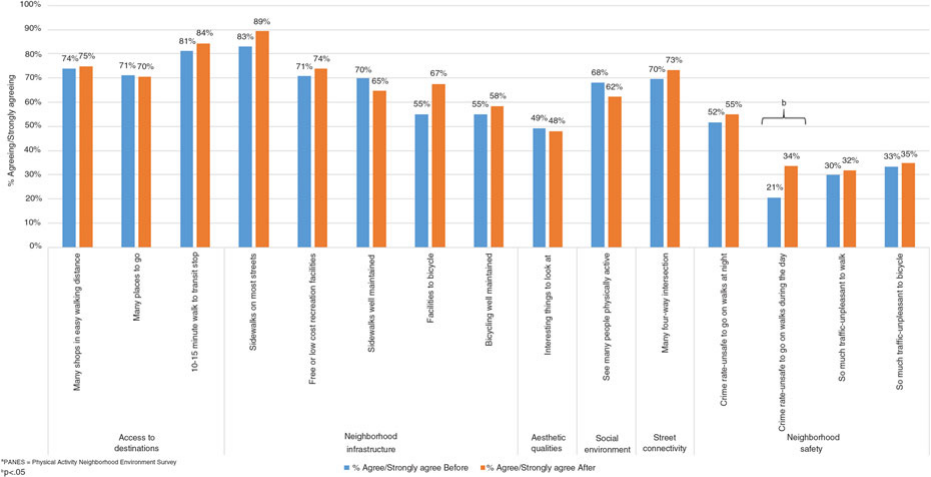
Percent of participants agreeing or strongly agreeing with items from the PANES before and after August 9, 2014, St. Louis, MO.

The percent of participants agreeing with the PANES statements is presented in Figure 1. Table 2 includes the frequency and percent of participants agreeing or disagreeing with the statements and the full item wording for each statement. Most (*n*=152, 63%) participants reported living in neighborhoods with a majority of multifamily housing (e.g., town houses, row houses, apartments, or condos; mix of single-family and multifamily residences; or apartments or condos). Among all baseline participants combined, availability of sidewalks (86%) and low-cost recreation facilities (72%) was high; however, fewer (67%) respondents agreed that the sidewalks were well maintained. Respondents reported that safety in their neighborhood was limited by several features such as the crime rate at night (53%), the crime rate in the day (27%), and traffic (31%).

**Table 2.** Participant Perceptions of the Neighborhood Environment Attributes and Associations Between Timing of the Baseline Survey (Before or After August 9, 2014, the Date Michael Brown Was Shot) and Agreement with the Statement Both Crude and Adjusted, St. Louis, MO

	Before (referent)/after	Disagree/SD, n (%)	Agree/SA, n (%)	Chi-square, p	Total	Crude OR (95% CI)^a^	Adjusted OR (95% CI)^a,b^
Access to destinations							
Many shops, stores, markets, or other places to buy things I need are within easy walking distance to my home	Before	34 (26.2)	96 (73.9)		130	1.05 (0.60–1.83)	1.06 (0.60–1.87)
	After	33 (25.2)	98 (74.8)	0.859	131		
	Overall	67 (25.7)	194 (74.3)		261		
There are many places to go within easy walking distance of my home	Before	38 (28.8)	94 (71.2)		132	0.96 (0.57–1.64)	0.95 (0.56–1.64)
	After	39 (29.6)	93 (70.5)	0.892	132		
	Overall	77 (29.2)	187 (70.8)		264		
It is within a 10–15-min walk to a transit stop (such as a bus, train, or tram) from my home	Before	24 (18.8)	104 (81.3)		128	1.25 (0.65–2.39)	1.20 (0.62–2.33)
	After	20 (15.6)	108 (84.4)	0.508	128		
	Overall	44 (17.2)	212 (82.8)		256		
Neighborhood infrastructure							
There are sidewalks on most of the streets in my neighborhood	Before	22 (16.9)	108 (83.1)		130	1.70 (0.83–3.50)	1.71 (0.83–3.54)
	After	14 (10.7)	117 (89.3)	0.144	131		
	Overall	36 (13.8)	225 ()86.2		261		
My neighborhood has several free or low-cost recreation facilities, such as parks, walking trails, bike paths, recreation centers, playgrounds, and public swimming pools.	Before	36 (29.3)	87 (70.7)		123	1.17 (0.67–2.03)	1.17 (0.66–2.07)
	After	33 (26.2)	93 (73.8)	0.588	126		
	Overall	69 (27.7)	180 (72.3)		249		
The sidewalks in my neighborhood are well maintained (paved, with few cracks) and not obstructed	Before	38 (30.2)	88 (69.8)		126	0.80 (0.47–1.35)	0.85 (0.50–1.44)
	After	45 (35.2)	83 (64.8)	0.396	128		
	Overall	83 (32.7)	171 (67.3)		254		
There are facilities to bicycle in or near my neighborhood, such as special lanes, separate paths or trails, shared use paths for cycles and pedestrians	Before	50 (45.1)	61 (55.0)		111	1.70 (1.00–2.89)	**1.76** (**1.02–3.04**)
	After	40 (32.5)	83 (67.5)	0.049	123		
	Overall	90 (38.5)	144 (61.5)		234		
Places for bicycling (such as bike paths) in and around my neighborhood are well maintained and not obstructed	Before	49 (45.0)	60 (55.1)		109	1.15 (0.68–1.93)	1.22 (0.72–2.06)
	After	52 (41.6)	73 (58.4)	0.605	125		
	Overall	101 (43.2)	133 (56.8)		234		
Aesthetic qualities							
There are many interesting things to look at while walking in my neighborhood	Before	65 (50.8)	63 (49.2)		128	0.96 (0.59–1.56)	0.99 (0.60–1.64)
	After	68 (51.9)	63 48.1 ()	0.856	131		
	Overall	133 (51.4)	126 (48.6)		259		
Social environment							
I see many people physically active in my neighborhood doing things such as walking, jogging, cycling, or playing sports and active games	Before	41 (31.8)	88 (68.2)		129	0.77 (0.46–1.29)	0.80 (0.48–1.34)
	After	50 (37.6)	83 (62.4)		133		
	Overall	91 (34.7)	171 (65.3)	0.323	262		
Street connectivity							
There are many four-way intersections in my neighborhood	Before	37 (30.3)	85 (69.7)		122	1.19 (0.69–2.07)	1.18 (0.67–2.05)
	After	34 (26.8)	93 (73.2)	0.534	127		
	Overall	71 (28.5)	178 (71.5)		249		
Neighborhood safety							
The crime rate in my neighborhood makes it unsafe to go on walks at night^c^	Before	59 (48.4)	63 (51.6)		122	1.14 (0.69–1.89)	1.16 (0.69–1.93)
	After	55 (45.1)	67 (54.9)	0.608	122		
	Overall	114 (46.7)	130 (53.3)		244		
The crime rate in my neighborhood makes it unsafe to go on walks during the day^c^	Before	100 (79.4)	26 (20.6)		126	**1.95** (**1.10–3.43**)	**2.05** (**1.15–3.67**)
	After	85 (66.4)	43 (33.6)	**0.020**	128		
	Overall	185 (72.8)	69 (27.2)		254		
There is so much traffic on the street that it makes it difficult or unpleasant to walk in my neighborhood^c^	Before	89 (70.1)	38 (29.9)		127	1.09 (0.65–1.85)	1.17 (0.68–2.00)
	After	90 (68.2)	42 (31.8)	0.741	132		
	Overall	179 (69.1)	80 (30.9)		259		
There is so much traffic on the streets that it makes it difficult or unpleasant to ride a bicycle in my neighborhood^c^	Before	78 (66.7)	39 (33.3)		117	1.07 (0.63–1.82)	1.12 (0.65–1.93)
	After	84 (65.1)	45 (34.9)	0.798	129		
	Overall	162 (65.9)	84 (34.1)		246		
		Detached single-family housing, *n* (%)	Multifamily homes, *n* (%)				
Residential density							
What is the main type of housing in your neighborhood?	Before	46 (38.3)	74 (61.7)		120	0.89 (0.53–1.50)	0.91 (0.54–1.55)
	After	43 (35.5)	78 (64.5)	0.653	121		
	Overall	89 (36.9)	152 (63.1)		241		

Bold indicates *p* < 0.05.

^a^ OR=odds ratio and CI.

^b^ Maternal age, income, education, marital status.

^c^ Reverse coded for neighborhood environment index.

CI, confidence interval.

When comparing neighborhood perceptions between those who responded to the baseline survey before August 9, 2014, and those who completed the survey after, differences were not observed in the main type of housing, access to destinations, aesthetic qualities, social environment, or street connectivity (Table 2). There was a marginal statistically significant difference for access to bicycle facilities, with a greater percent of those completing the survey after August 9, 2014, agreeing “There are facilities to bicycle in or near my neighborhood…” (55% before, 68% after, *p*=0.049), although the odds ratio (1.8) was only significant in the adjusted model. While three of the four neighborhood safety items (crime at night, traffic for biking, and traffic for walking) were not different between those completing the survey before compared with after August 9, 2014; 34% of the participants completing the survey after this date felt “The crime rate in my neighborhood makes it unsafe to go on walks during the day” compared with 21% of participants who completed the survey before (adjusted odds ratio: 2.0, 95% confidence interval: 1.1–3.7).

## Discussion

Compared with mothers who completed surveys before August 9, 2014, a greater percent of mothers completing the survey after the death of Michael Brown reported feeling unsafe in their neighborhood during the day. Perceptions of many other neighborhood features related to physical infrastructure such as number of four-way intersections and availability of sidewalks were not different before and after.

The difference in perceived crime rate highlights the potential for local events to influence neighborhood perceptions related to health behaviors. That, as mentioned above, there were no other consistent differences between the mothers completing the survey between these time points strengthens the confidence that the difference in perceptions of the crime rate may have been related to the events not just of the initial shooting but those that followed in the subsequent weeks and months. This may include the civic unrest and the growing mistrust in law enforcement. These differences are in line with a panel survey of St. Louis County residents conducted by Kochel,^13^ which included four administrations, before the shooting (March to May 2012, November 2012 to January 2013, and May to July 2013) and one immediately after (September and October 2014). The findings of the panel study demonstrate increases in residents reporting seeing aggressive policing and decreases in a sense of procedural justice and police legitimacy among African American residents. Others have hypothesized that events such as those in Ferguson, Missouri, can lead to perceptions of a decline in police presence out of fear of community distrust, which may be another reason for participants to report feeling unsafe.^26–28^ The findings of the current study also demonstrate the sensitivity of the PANES measure to pick up on a change in the community context when external unexpected social forces are experienced by individual community members. This can help researchers and practitioners understand local events and pressures, and lead to interventions that are able to take these into account.

As has been widely discussed, efforts to promote health and achieve health equity must take social determinants of health into consideration. This study is an example of how an acute shift in community context beginning with a profound event can be detected with repeated assessments of individual perceptions of their neighborhood in ways that can impact health behaviors and health outcomes. Throughout this period in the intervention study from which these baseline data are drawn, mothers were interacting with study interventionists and participating in the lifestyle change intervention promoting health behavior changes. Given the length of the intervention (from 15 weeks pregnant until 18 months postpartum), issues related to these events frequently arose in both planning intervention visits (the study involved home visits) and the ability to incorporate the suggested health behaviors.^25^ While studies at the national level may inquire about neighborhood environmental features or try to take social determinants of health into account, this may be difficult without the knowledge of current events in local communities and how participants experience these events.

Impactful events and changing contexts are particularly important for interventions deigned to be sustained over months and years (e.g., the 12-month Diabetes Prevention Program^29^) and to promote health behaviors in ways that can be maintained throughout the life-course. Furthermore, in determining who might be appropriate interventionists, an important consideration might be cultural congruence, and selection of individuals with ties to and deep knowledge of the communities in which they will work and local events that affect those communities, and the skills to understand and incorporate these contextual factors. To achieve health equity, studies should at least assess local events at the outset, but must also incorporate this dynamic context into the intervention. For example, offering intervention interactions by text message, when participants do not feel safe leaving their home or dedicated intervention visit time to helping participants process local events, rather than focusing on predetermined health behaviors.

The current study adds to the literature of studies using the PANES measure by reporting on perceptions among a group of underserved, pregnant African American women in a midwestern city in the United States. There were notable differences for several neighborhood perception items in this population and setting compared with other contexts where PANES has been used, particularly regarding safety. For example, perceptions that the crime rate made it unsafe to go on walks during the day were lower in a study by Sallis et al. (15%)^19^ than among mothers in the current study (21% before August 9, 2014, and 34% after). The participants surveyed by Sallis et al. were primarily white and with incomes >$50,000. The current study represents a unique group of participants to have completed the PANES measure, as all women identified as African American and a majority reported income <$10,000.

The findings of this study should be interpreted with limitations in mind. The PANES measure is self-reported and does not include objective assessments of the environment; however, the focus of the measure is on perception of the neighborhood environment, rather than an objective assessment as environmental perception likely has important influences on behavior.^30^ The study is similarly limited to neighborhood perceptions, rather than activity behaviors. Measures of activity would provide a deeper understanding of how the events may influence not only perceptions but also behavior. It may be, however, that the perceptions detected in the current survey point to experiences that led women to alter their lives in ways unrelated to physical activity.

In addition, follow-up studies utilizing qualitative methods may allow for a richer understanding of their experience, which is missed in this quantitative study. Such qualitative methods could also have captured critical issues related to neighborhood safety, before, during, and after the unrest in Ferguson, Missouri; as a quantitative study, the current analyses are not able to provide a full description of the participants' experiences. Furthermore, we did not find a significant association with crime rate during the night, which calls into question the face validity of our finding that the perceptions of crime during the day were significantly associated with timing. However, this group of mothers (both those taking the survey before and those after) reported lower safety at night than participants completing the same survey in other studies^19^; therefore, there may have been little difference before and after the events.

In addition, the comparison in the current study is nonrandom. Confounding control is a particular challenge in unplanned, nonexperimental studies of this sort. Our study simultaneously underscores the importance of looking for unexpected environmental factors that might influence a public health study, and the statistical limitations (i.e., the threats to internal validity resulting from a not experimental design such as history effects and the potential for unmeasured confounders) that constrain our ability to rigorously examine those factors. This study takes advantage of data already collected to build an initial understanding of this very important community event, while acknowledging these important limitations. Any findings yielded from such practical analysis will likely require more intentional and directed follow-up. Although the lack of significant differences between participants completing the survey before and after August 9, 2014, suggests there are not systematic differences, it is not possible to rule out other causes of the association. The study cannot explain why the events may have led to differences in the percent of women reporting feeling unsafe, although there are many hypotheses around how events such as those in Ferguson, Missouri, may be related to changes in safety perceptions and police relations.^26,31,32^ Finally, there is a wide range of time between when participants completed the survey and August 9, 2014, meaning some women took the survey just days before and others just days after, while some women were more than a year away. Given that the St. Louis community continues to struggle with disparities and the aftermath of these events, it may be that the experience resonated with participants even over many months.

### Health equity implications

This study demonstrates empirically how events in a community can influence residents' perceptions, and how those perceptions may be related to important social determinants of health (e.g., safety of environment). While appreciation of the importance of social determinants of health is recognized, the complex and nuanced nature of intervening on these determinants is daunting for many, including researchers and practitioners who develop, implement, and evaluate interventions. Such professionals must grapple with this complexity, for example, by building interventions that adapt to a participant's needs in the wake of an unexpected change in social context, and by looking for the impacts of such changes in the evaluation and interpretation of research. This also highlights the importance of building community partnerships and community-led research as well as a trauma-informed approach to research.^33,34^ Ultimately, achieving health equity requires that interventions to reduce disparities, promote health behaviors, and improve health outcomes consider not just the environments where people live and work, but the dynamic nature of those environments as well.

## Abbreviations Used

CI: confidence interval
PANES: Physical Activity Neighborhood Environment Survey
SD: standard deviation
